# Multiple Sclerosis Presents with Psychotic Symptoms and Coexists with Hypertrophic Cardiomyopathy

**DOI:** 10.1155/2014/383108

**Published:** 2014-08-13

**Authors:** Muhammed Emin Özcan, Bahri İnce, Hasan Hüseyin Karadeli, Talip Asil

**Affiliations:** ^1^Department of Neurology, Bezmialem Vakif University Medical School, Adnan Menderes Boulevard, Fatih, 34093 Istanbul, Turkey; ^2^Department of Psychiatry, Bakirkoy Research and Training Hospital for Psychiatry, Bakirkoy, 34147 Istanbul, Turkey

## Abstract

Multiple sclerosis (MS) is a demyelinating disease of the central nervous system. Psychiatric symptoms are not infrequent during MS, yet onset of MS with psychosis is rarely encountered. A 27-year-old Caucasian male was admitted due to numbness in his right arm and difficulty in walking. His clinical and laboratorial exams lead to the MS diagnosis. Nine months earlier, he also developed psychotic disorder, not otherwise specified (PD-NOS). His sudden onset of PD-NOS, his rapid and complete response to antipsychotics, and a relatively short interval between psychiatric and neurological signs indicate a high likelihood that PD-NOS was a manifestation of underlying MS. He also suffers from hypertrophic obstructive cardiomyopathy (HOCM). The patient's neurological complaints were recovered with methylprednisolone (1 g/day, i.v.) given for five days. Glatiramer acetate (1 × 1 tb.s.c.) was prescribed for consolidation and, after nine months of his admission, the patient fully recovered from neurological and psychiatric complaints. Interestingly, very recent studies indicate specific alpha-actinin antibodies in MS and alpha-actinin mutations cause HOCM. Thus, concurrence of MS with HOCM can be even a new syndrome, if further genetic studies prove.

## 1. Introduction

Multiple sclerosis (MS) is an inflammatory demyelinating disease of the central nervous system. Concomitant psychiatric diseases are frequent in MS [1] and, among these, depression and anxiety disorders constitute the highest percentages. Psychotic disorders accompanying MS occur less frequently with rates of 2-3% [2]. Psychotic symptoms usually develop after the neurological signs and they are mostly linked to the side effects of interferon beta-group immune modifiers [3]. The onset of MS with isolated psychotic symptoms is rare and cases which first develop psychotic signs without neurological deficits and triggering stressors are highly limited [4, 5]. Hypertrophic obstructive cardiomyopathy (HOCM) is a rare genetic disorder characterized by left ventricular outflow tract obstruction. Concurrence of MS and HOCM is extremely rare. In this report, we present a 27-year-old male patient who developed psychotic symptoms nine months prior to MS.

## 2. Case Presentation

**Figure 1:**
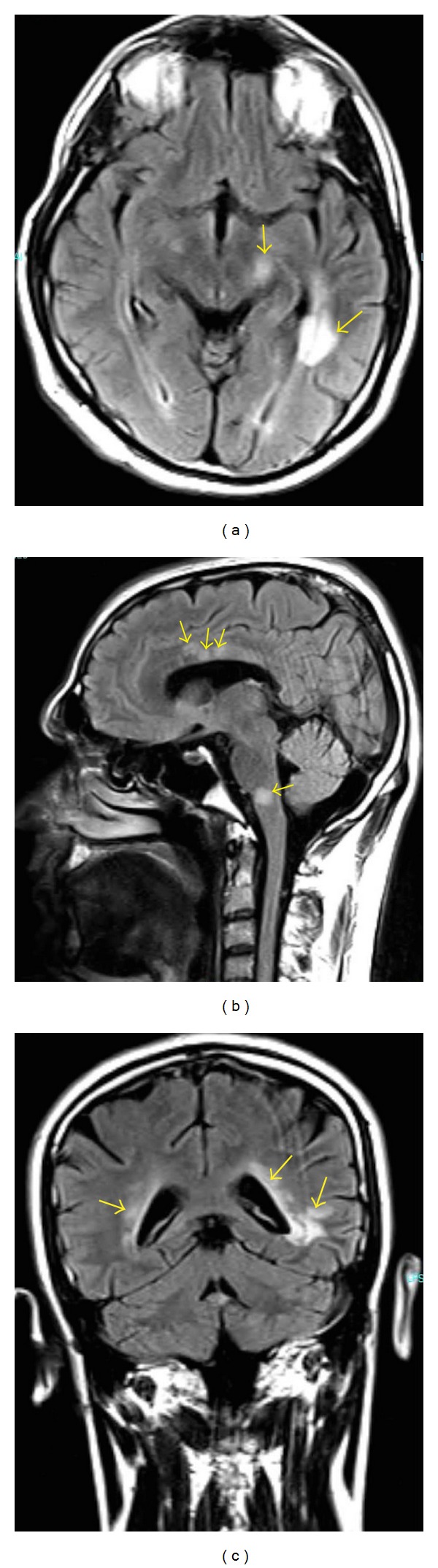
Cranial MRI showing (a) axial, (b) sagittal, and (c) coronal T2-weighted FLAIR views of the demyelinating lesions in the bilateral periventricular, juxtacortical, and infratentorial regions (arrows).

**Figure 2:**
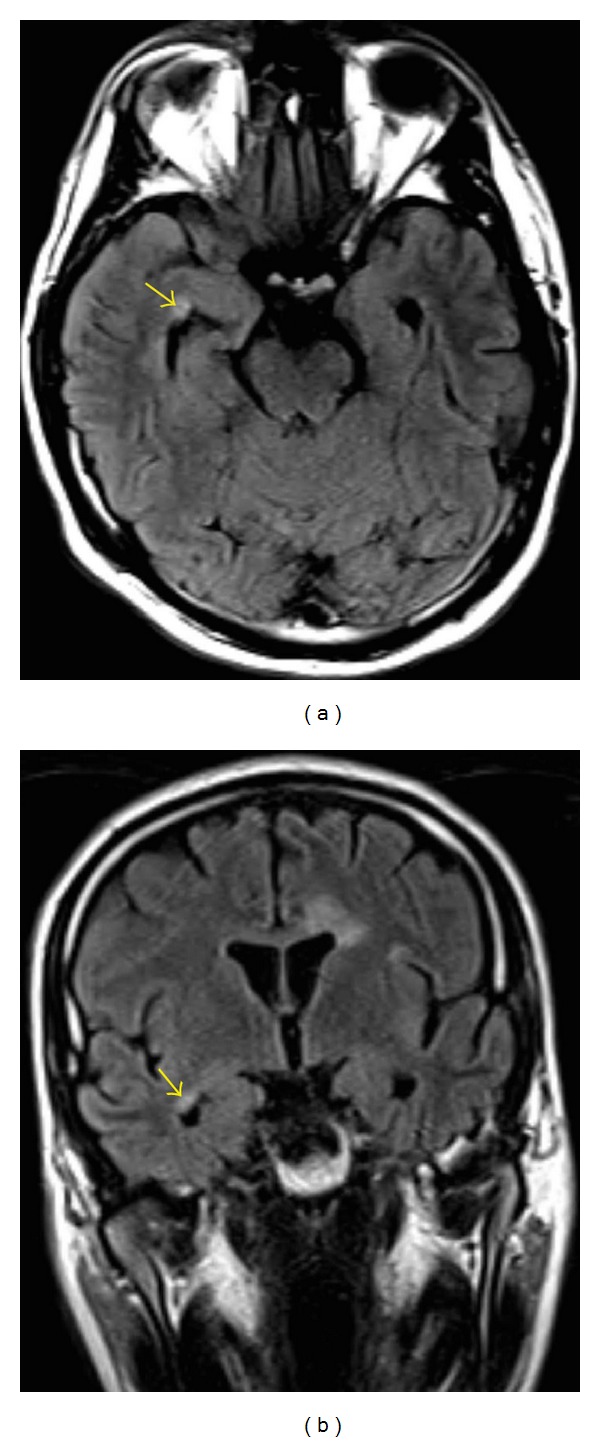
Follow-up cranial MRI showing (a) axial and (b) coronal T2-weighted FLAIR view of the new demyelinating lesion in the right temporal white matter (arrows).

A 27-year-old male patient was admitted to our neurology department with numbness in the right arm and difficulty in walking for three days. Nine months earlier, he developed beliefs that he was threatened by his colleagues to marry a woman and that everyone made plans for him. Furthermore, the patient remarked that he had seen snakes in his room and felt extremely frightened. Then, the patient was evaluated by different psychiatrists for 7 months, yet irregularly due to lack of the patient's compliance. The patient was first prescribed olanzapine (10 mg, b.i.d. po.) with the diagnosis of PD-NOS and he benefited from this treatment. However, he discontinued after four weeks, and a similar, yet more severe, state reemerged ten days after his cessation. Then, he was admitted to our psychiatry clinic and continuation of olanzapine was recommended. His complaints regressed swiftly and he returned to work. During his followup, he said that he again discontinued olanzapine due to excessive weight gain. Thus, he was prescribed paliperidone (6 mg, q.d. po.) and he underwent remission. The patient also suffers from HOCM and septal ablation was applied in 2012 due to arrhythmia. There were no unusual features in his family history and he did not report usage of psychoactive substances. At neurological examination, orientation, consciousness, and cooperation were normal; speech was normal; vision was 20/20 in both eyes; eye movements were normal in all directions; and other cranial nerves, brain stem functions, and bladder and bowel functions were normal. Muscle strength in right upper extremity was 4/5 and was normal in the other extremities. Deep tendon reflexes were hyperactive in the upper and lower extremities, with bilateral extensor plantar responses. Sensory examination was normal except for a subjective decrease in sensation to pinprick in the right upper extremity. Cranial MRI demonstrated hyperintense, noncontrast enhancing demyelinating lesions on T2 and FLAIR images in the bilateral periventricular and left temporal lobe white matter, the corpus callosum, and the mesencephalon (Figure 1). Cervical and thoracic spinal MRI was normal. The patient was hospitalized. Biochemical markers were negative for lupus and rheumatism disorders. Negative pathergy test and lack of aphthae excluded Behçet syndrome, common in Turkey. Infection markers for Lyme disease, brucellosis, CMV, and HS viruses were negative. Microscopy and biochemistry of the cerebrospinal fluid (CSF) were normal. Isoelectric focusing revealed presence of the oligoclonal band in the CSF. The patient was administered methylprednisolone (1 g/day, i.v.) for 5 days and, thereafter, complaints in his right arm were resolved except for partial hypoesthesia. The consultant psychiatrist recommended continuation of paliperidone. One month following discharge, cranial MRI revealed new T2 and FLAIR hyperintense lesion in the right temporal white matter (Figure 2). The patient was prescribed glatiramer acetate (20 mg. q.d. sc.). There were no neurological or psychiatric findings nine months after his admission.

## 3. Discussion

The patient's neurological symptoms, when considered together with MR findings, lead to the clinically isolated syndrome diagnosis. One month following discharge, cranial MRI revealed new T2 and FLAIR hyperintense lesion in the right temporal white matter. One clinical attack, demonstration of dissemination in space (minimum of 1 T2 hyperintense lesion should be present in at least 2 of 4 characteristic locations: periventricular, juxtacortical, infratentorial, and/or spinal cord), and dissemination in time (a new T2 and/or gadolinium-enhancing lesion(s) on follow-up MRI, irrespective of its timing with reference to a baseline scan) lead to the diagnosis of multiple sclerosis according to the McDonald 2010 diagnostic criteria [6].

The prevalence of neuropsychiatric symptoms in MS can vary according to various series; some authors report levels as high as 95% [2]. Depressive symptoms are most frequent among these. Psychotic symptoms are rare as initial findings of MS. The current case presents a psychosis with reference and persecution delusions and visual hallucinations nine months prior to MS. One may propose that such an association emerged only due to incidental addition of a primary psychotic disorder; however several features indicate otherwise. One of these is the sudden onset of psychotic signs without prodromal period or loss of function. Another feature is that the neurological signs of MS appeared in a relatively short interval after the psychosis. Furthermore, live visual hallucinations and rapid response to antipsychotics support the organic etiology. In the literature, psychotic findings during MS are particularly associated with left temporal lobe T2 and FLAIR hyperintense lesions [7, 8]. The demyelinating lesions presented in our case are intensely observed in the left temporal lobe, which is accordant with the literature. The current case represents a good example of the necessity for a detailed examination of psychosis with sudden onset, and performing a cranial MRI has particular importance.

Atypical antipsychotics, particularly olanzapine, risperidone, and ziprasidone, are preferred for the psychosis accompanying MS [9]. The patient's comorbidity with HOCM also deserves further interest. An important gene mutation causing HOCM occurs in the locus of alpha-actinin [10], and its protein product is present in the heart and skeletal muscle and in the central nervous system. A recent study demonstrated that MS autoantibodies are specifically linked to alpha-actinin [11]. Considering these, comorbidity of MS and HOCM may also pave the way for finding novel etiological clues for both diseases. Lastly, in cases with concomitant MS and psychosis, it should be taken into account that both interferon-beta and high dose steroids may worsen psychosis and treatment strategies should be envisaged accordingly.
